# Identification and Validation of Reference Genes for Quantitative Real-Time PCR in *Ficus carica*

**DOI:** 10.3390/plants15010040

**Published:** 2025-12-22

**Authors:** Masahito Nakano

**Affiliations:** Toyo Institute of Food Technology, Kawanishi-shi 666-0026, Japan; masahito_nakano@shokuken.or.jp

**Keywords:** *Ceratocystis ficicola*, *Ficus carica*, *Gilbertella persicaria*, *Neofusicoccum* sp., reference gene, RT-qPCR

## Abstract

Fig (*Ficus carica* L.), a deciduous fruit tree that belongs to the *Moraceae* family, is cultivated worldwide as an important fruit crop for raw and processed foods. Quantitative real-time PCR (RT-qPCR) is a widely used method in *F. carica* to elucidate expression of genes related to various physiological responses. However, no studies have identified appropriate reference genes for RT-qPCR normalization in *F. carica*. In this study, 12 genes were selected from the *F. carica* genome as candidate reference genes for normalizing target gene expression. All candidate genes exhibited high amplification efficiency and specificity in the absence of primer dimers or extra PCR amplicons. The expression levels of the candidate genes were measured in three different plant tissues (fruit, leaf, and stem) under fungal pathogen infection using RT-qPCR. Their expression stabilities were evaluated using four computational algorithms: geNorm, Normfinder, delta-Ct, and BestKeeper. The RefFinder program was also used to calculate the geometric mean of the stability rankings obtained from these algorithms. The comprehensive ranking revealed that *FcYLS8*, *FcPP2A*, and *FcAP2M* were the most stable reference genes under biotic stress in the fruits, leaves, and stems, respectively. In contrast, traditional reference genes such as *FcACT2*, *FcEF-1α*, *FcGAPDH*, *FcUBC21*, and *FcUBQ5* exhibited relatively low expression stability in all tested tissues. This study identified and validated stable reference genes for RT-qPCR normalization in *F. carica*, thus providing a valuable resource for accurate gene expression studies under biotic stress and highlighting the importance of validating reference genes to ensure reliable and reproducible RT-qPCR analysis.

## 1. Introduction

Gene expression analysis is an important strategy in molecular biology for elucidating the complex signaling and metabolic networks underlying cellular and developmental processes. Quantitative real-time PCR (RT-qPCR) is considered a highly reliable method owing to its sensitivity, specificity, and reproducibility [1]. The reliability of RT-qPCR analysis is influenced by various experimental conditions, such as RNA quality, presence of inhibitors, and efficiency of RT and PCR [2]. In addition, the selection of endogenous reference genes is crucial for obtaining reliable RT-qPCR results, as it controls for biological and experimental errors across different samples.

The most frequently used reference genes in plants include 18S ribosomal RNA (*18S rRNA*), actin (*ACT*), elongation factor-1 (*EF-1*), glyceraldehyde-3-phosphate dehydrogenase (*GAPDH*), tubulin (*TUB*), ubiquitin-conjugating enzyme (*UBC*), and polyubiquitin (*UBQ*). However, these traditional reference genes do not consistently exhibit stability when applied to different experimental conditions [3,4], and their use for normalization can result in inaccurate results in gene expression analysis. In the post-genomic era, genomic and transcriptomic techniques have been adapted to survey more robust genes with minimal variability in *Arabidopsis thaliana* [3,5] and identified novel reference genes such as adaptor protein-2 μ-adaptin (*AP2M*), eukaryotic translation initiation factor 4A-1 (*EIF4A1*), F-box protein (*F-box*), protein phosphatase 2A (*PP2A*), SAND family protein (*SAND*), TIP41-like protein (*TIP41*), and yellow-leaf-specific protein 8 (*YLS8*). Recent studies have validated the expression profile and stability of orthologous genes to normalize transcription levels in agronomically important crops [6,7].

Fig (*Ficus carica* L.) is a deciduous fruit tree that belongs to the *Moraceae* family. It has been cultivated as an important fruit crop since ancient times, particularly in the Mediterranean region. Fig fruits are used as raw and processed foods as well as medicinal plants. Changes in color and sugar content during fruit development are important factors in the breeding of *F. carica*, and understanding their molecular mechanisms is necessary to improve fruit quality. Studies on gene expression analysis of fig fruits have recently been conducted to elucidate the molecular mechanisms involved in various physiological responses, including fruit development, coloring, and maturation [8,9,10]. The peels of dark-colored fig cultivars exhibit high levels of anthocyanin, with a corresponding increase in the expression of genes related to anthocyanin biosynthesis. In most RT-qPCR analyses of *F. carica*, *ACT* has been used as a reference gene [8,11,12]. Additionally, other studies have used *TUB*, *EF-1*, and *18S rRNA* [13,14,15]. Although these traditional reference genes have been extensively used in RT-qPCR analysis, the stability of their expression has not been evaluated in *F. carica*, raising concerns regarding their use for normalization in gene expression analysis. Therefore, selecting appropriate reference genes with minimal variability under certain experimental conditions is necessary for accurate RT-qPCR analysis of gene expression.

This study aimed to identify and validate suitable reference genes using RT-qPCR analysis in three tissues (fruit, leaf, and stem) of *F. carica* inoculated with fungal pathogens. A total of 12 candidate reference genes (*FcACT2*, *FcAP2M*, *FcEF-1α*, *FcEIF4A1*, *FcF-box*, *FcGAPDH*, *FcPP2A*, *FcSAND*, *FcTIP41*, *FcUBC21*, *FcUBQ5*, and *FcYLS8*) were selected from the *F. carica* genome, and the stability rankings of their expression levels were calculated in each tissue using four computational algorithms (geNorm, Normfinder, delta-Ct, and BestKeeper). Additionally, the RefFinder program was used to calculate the geometric mean of the stability rankings derived from these algorithms. Based on the comprehensive rankings, this study provides a set of stable reference genes for normalizing transcription levels under biotic stress in *F. carica* and underscores the importance of validating reference genes to ensure reliable and reproducible RT-qPCR analysis.

## 2. Materials and Methods

### 2.1. Plant Material and Growth Conditions

Fig (*F. carica* L. cv. San Piero) was grown in a controlled-environment room at 24 °C with a photoperiod of 14 h of light (12,000 lux) and relative humidity of 40%. Two-year-old trees were used for the experiments.

### 2.2. Total RNA Extraction and cDNA Synthesis

For total RNA extraction, fruit, leaf, and stem tissues of *F. carica* were collected from healthy plants at the same developmental stage. Fruit tissues were sampled from fully mature fruits, leaf tissues were excised from fully expanded fifth leaves below the shoot apex, and stem tissues were collected from young green shoots by cutting small segments with a sterile scalpel. All tissues were immediately frozen in liquid nitrogen and stored at −80 °C until use.

cDNA preparation was performed as previously described [16] with slight modifications. Total RNA was extracted from 30 mg *F. carica* tissue using 1 mL RNAiso Plus reagent (Takara, Kusatsu, Japan). RNA was purified using the NucleoSpin RNA Plant kit (Takara), and cDNA was synthesized with 1 μg RNA using a PrimeScript FAST RT reagent kit with gDNA Eraser (Takara) according to the manufacturer’s instructions.

### 2.3. Cloning of Reference Genes

Full-length sequences of A. thaliana reference genes were obtained from the TAIR database (released on 9 April 2024) and used as queries for BLAST v2.16.0 search against the *F. carica* genome (GenBank ID: BTGU00000000.1; Assembly ID: GCA_033242285.1) using the default parameters (expect threshold, 0.05; word size, 5; matrix, BLOSUM62) to identify orthologous genes. To confirm the accuracy of the gene sequences obtained from the BLAST search, the coding region of the top-scoring *F. carica* genes was PCR-amplified using primer sets (Table S1) and inserted into the pCRII-TOPO vector (Thermo Fisher Scientific, Waltham, MA, USA). Eurofins Genomics Inc. (Tokyo, Japan) sequenced the regions inserted in the resultant vectors, and sequence data analysis was performed using GENETYX software v15.0.3 (Nihon server, Tokyo, Japan).

### 2.4. Quantitative PCR Analysis

Gene-specific primers for RT-qPCR analysis were designed using Primer3Plus program v3.3.0 [17] with the following parameters: product size range, 80–150 bp; primer size, 17–25 bp; primer Tm, 60–65 °C; primer GC, 45–55%. Eurofins Genomics Inc. synthesized the primer sets used for RT-qPCR analysis (Table 1).

Quantitative PCR was conducted as previously described [16] with slight modifications. The reaction mixture contained 10 μL TB Green Premix Ex Taq II (Takara), 0.4 μM of each gene-specific primer, 0.4 μL ROX Reference Dye II, and 1 μL cDNA in a total volume of 20 μL. The PCR reaction was performed on a QuantStudio 3 real-time PCR system (Thermo Fisher Scientific) with the following thermocycling conditions: 1 cycle of 95 °C for 30 s, 40 cycles of 95 °C for 3 s, and 60 °C for 30 s. Melting curves were recorded at the end of the amplification reaction by heating from 60 to 95 °C at a ramp speed of 1 °C min^−1^. After the RT-qPCR reaction, the amplification products were electrophoresed using a D1000 ScreenTape (Agilent Technologies, Santa Clara, CA, USA) on an automated electrophoresis system TapeStation 4150 (Agilent Technologies) according to the manufacturer’s instructions. The expression level of *FcPR1* was normalized to those of multiple endogenous control genes, such as *FcYLS8*, *FcPP2A*, *FcAP2M*, *FcEF-1α*, and *FcGAPDH*.

For the amplification efficiency and correlation coefficient, serial ten-fold dilutions of the *F. carica* cDNA pool (1–1000 dilutions) derived from different tissues were used as the template for RT-qPCR. The amplification efficiency (E) and correlation coefficient (R^2^) of each reference gene were calculated using the following equation: E (%) = (10^−1/slope^ − 1) × 100. In this equation, slope was derived from the linear regression analysis.

### 2.5. Pathogen Growth Conditions and Inoculation

The fungal pathogens *Gilbertella persicaria* SHK1000 [18], which causes soft rot of fig fruit, *Neofusicoccum* sp. FFNP0901 (MAFF243818), which causes black leaf blight of fig leaf, and *Ceratocystis ficicola* FFCF9001 [19], which causes canker of fig stem, were cultured on potato dextrose agar (PDA) medium for 3 days, 2 weeks, and 3 weeks, respectively. Inoculation experiments were conducted on detached mature fruits, fully expanded leaves, and young vegetative shoots. For *G. persicaria*, unwounded fig fruits were sprayed with a spore suspension (1 × 10^6^ spores mL^−1^) or sterile water as a negative control [18]. For *Neofusicoccum* sp., a pinhole was made in the leaves using a needle and covered with either a mycelium plug (5 mm in diameter) or a PDA plug as a negative control. For *C. ficicola*, a wound (6 mm in diameter) was created on the outer bark of the shoots using a cork-borer and challenged by inserting a mycelium plug or a PDA plug as a negative control into the wound [20]. The wound areas were covered with parafilm to prevent desiccation and contamination. The inoculated plant tissues were incubated in a moist chamber at 25 °C in the dark. Each tissue (30 mg) was harvested for total RNA isolation 1 day after inoculation for *G. persicaria*, 2 days after inoculation for *Neofusicoccum* sp., and 1 week after inoculation for *C. ficicola*.

### 2.6. Data Analysis

The expression levels of the reference genes were determined by the number of amplification cycles (Cq). The expression stability of each gene was evaluated using four computational algorithms (geNorm, Normfinder, delta-Ct, and BestKeeper) in the RefSeeker R package v1.0.4 [21]. All other statistical analyses were performed with GraphPad Prism software v8.4.3.

## 3. Results and Discussion

### 3.1. Selection of Candidate Reference Genes

To identify the most stable genes in *F. carica*, 12 orthologs were selected as candidate reference genes based on previously reported studies of plant reference genes. Of the 12 genes selected, five (*ACT2*, *EF-1α*, *GAPDH*, *UBC21*, and *UBQ5*) have been traditionally used as reference genes in *Arabidopsis thaliana*, and seven genes (*AP2M*, *EIF4A1*, *F-box*, *PP2A*, *SAND*, *TIP41*, and *YLS8*) were recently identified as the most reliable reference genes through genome-wide analysis. During the selection process of candidate reference genes, *18S rRNA* was excluded owing to its high abundance compared with that of the target mRNA transcripts, which complicates the accurate subtraction of baseline values in RT-qPCR data analysis [22]. Using the *A. thaliana* orthologous genes as query sequences for a homology search in the BLAST database, each candidate reference gene was isolated and identified from the *F. carica* cDNA pool prepared from the fruit, leaf, and stem tissues. The amino acid sequences of the reference genes showed relatively high identity with their *A. thaliana* orthologs, ranging from 72% for *FcTIP41* to 99% for *FcYLS8* (Table S1).

### 3.2. Primer Specificity and Efficiency of Candidate Reference Genes

The RT-qPCR primer sets were designed to target the 12 candidate reference genes (Table 1) and tested for their specificity in the RT-qPCR analysis using the *F. carica* cDNA pool prepared from the fruit, leaf, and stem tissues. The RT-qPCR analysis was conducted to verify the primer specificity for amplifying the candidate reference genes. In the melting curve analysis followed by RT-qPCR, each candidate gene exhibited a clear and single peak without primer dimer formation (Figure S1A). After RT-qPCR, the reaction mixtures were separated on an automated electrophoresis system. All primer sets provided a single PCR product with the expected amplicon length (Figure S1B).

The RT-qPCR efficiency was determined for each primer set by standard curve analysis using serial dilutions of the *F. carica* cDNA pool prepared from the fruit, leaf, and stem tissues. The efficiency of the primer sets ranged from 91.14% for *FcYLS8* to 108.33% for *FcACT2* (Table 1) and was within the acceptable range of 90–110%. The correlation coefficients of the primer sets varied from 0.996 to 0.999, meeting the criteria (>0.99) described in the RT-qPCR instrument manual (QuantStudio 3 real-time PCR system, Thermo Fisher Scientific).

Collectively, all the designed primer sets showed high specificity and efficiency for amplifying the reference genes. Therefore, the following analyses were performed using the primer sets designed in this study.

### 3.3. Expression Profiling and Stability of Candidate Reference Genes in Different Tissues Under Biotic Stress

**Figure 1:**
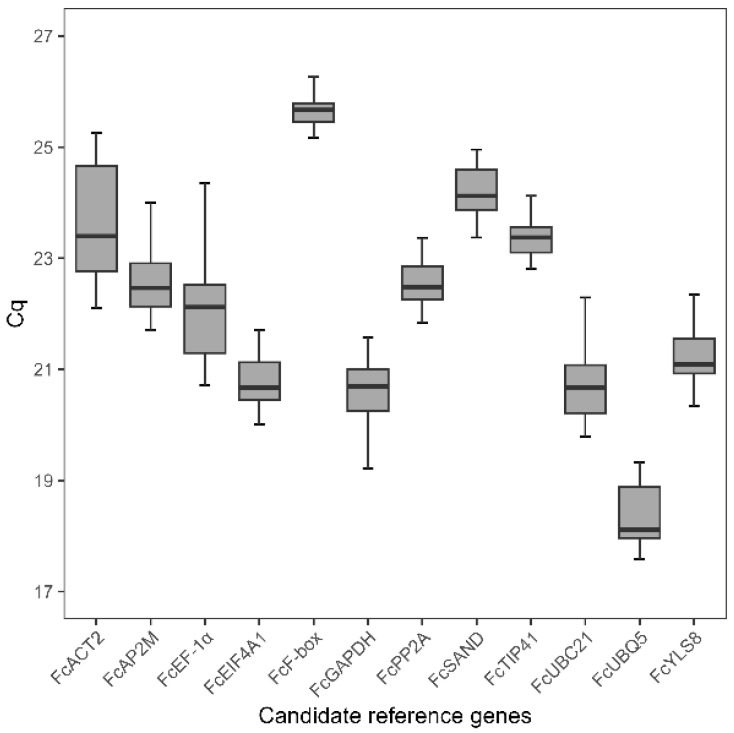
Expression profiles of candidate reference genes in different tissues of *Ficus carica* under biotic stress. Total RNA was isolated from the fruit, leaf, and stem tissues of *F. carica* inoculated with fungal pathogens or mock. The expression levels of 12 candidate reference genes were determined by RT-qPCR and are shown as raw Cq values in three tissues (fruits, leaves, and stems) for two inoculations (mock and pathogen) from three independent experiments, each with three biological replicates (54 cDNA samples). The box indicates the 25th and 75th percentiles, with the bold line indicating the median value. The whisker caps represent the minimum and maximum values.

The gene-specific primer sets were used to amplify each candidate reference gene using *F. carica* cDNA samples prepared from three tissues (fruits, leaves, and stems) for two treatments (mock and pathogen) from three independent experiments, each with three biological replicates (*n* = 54 for each gene). The gene expression levels were determined as Cq values, and the transcripts of the candidate genes exhibited different levels of abundance. The Cq values of each gene ranged from 17.58 to 26.91 (Figure 1), which is within the acceptable range of 15–30, as indicated in the general guidelines for qPCR [23]. Among the 12 genes tested, *FcF-box* exhibited the lowest expression, with a mean Cq value of 25.78, whereas *FcUBQ5* exhibited the highest expression, with a mean Cq value of 18.43. Most of the Cq values were distributed between 21 and 24. *FcF-box* and *FcTIP41* showed relatively low expression levels, with a narrow range of ΔCq values of 1.10 and 1.32, whereas the traditional reference genes such as *FcEF-1α*, *FcACT2*, and *FcUBC21* exhibited relatively wide variations, with ΔCq values of 3.89, 3.15, and 2.92, respectively. This result indicates that all 12 candidate genes showed a consistent expression level across the different plant tissues under biotic stress.

**Figure 2:**
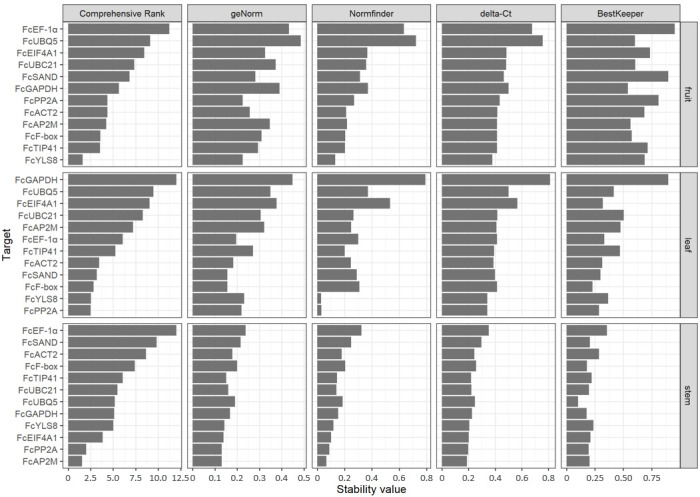
Stability ranking of candidate reference genes in different tissues of *Ficus carica* under biotic stress. Total RNA was isolated from the fruit, leaf, and stem tissues of *F. carica* inoculated with fungal pathogens or mock. The expression levels of 12 candidate reference genes were determined by RT-qPCR and are shown as raw Cq values in three tissues (fruits, leaves, and stems) for two inoculations (mock and pathogen) from three independent experiments, each with three biological replicates (54 cDNA samples). The expression stability values of each gene were evaluated in different tissues using four algorithms: geNorm, Normfinder, delta-Ct, and BestKeeper. The comprehensive rank was calculated from the individual rankings of the algorithms using the RefFinder program. Based on the comprehensive ranking order, the most stable genes are at the bottom, whereas the least stable genes are at the top.

To determine the optimal reference gene for RT-qPCR among the 12 candidate genes, four computational algorithms (geNorm, Normfinder, delta-Ct, and BestKeeper) were used to calculate the expression stability (M value) of each gene in three plant tissues (fruits, leaves, and stems). In geNorm, all candidate genes met the high expression stability criterion with M < 0.486, which is considerably below the accepted threshold of 1.5 [22]. The geometric mean of the stability rankings was calculated in different plant tissues using the RefFinder program based on the rankings order derived from the algorithms. Comprehensive ranking identified *FcYLS8*, *FcPP2A*, and *FcAP2M* as the most stable reference genes in fruit, leaf, and stem tissues, respectively (Figure 2). In fruits, *FcLYS8*, *FcTIP41*, and *FcF-box* were the top three reference genes with the highest stability. *FcPP2A*, *FcYLS8*, and *FcF-box* showed high expression stability in leaf tissues, while *FcAP2M*, *FcPP2A*, and *FcEIF4A1* showed higher stability in stem tissues.

Most RT-qPCR analyses in *F. carica* have extensively used the traditional reference genes *ACT* and *18S rRNA* to normalize target genes under various conditions, such as different environmental stress, plant cultivar, and tissue [8,11,12,14]. Notably, the traditional reference genes *FcACT2*, *FcEF-1α*, *FcGAPDH*, *FcUBC21*, and *FcUBQ5* demonstrated relatively low expression stability in all tested tissues and were not ranked within the top three on the comprehensive ranking (Figure 2). Recent studies suggest that traditional reference genes selected for normalization may be unsuitable because their status as reference genes is generally based on qualitative techniques such as Northern blot, RNase protection assay, and conventional RT-PCR, which are inconsistent with the high accuracy of RT-qPCR [2]. In various plant species, *18S rRNA* is highly abundant compared with other reference genes, and its expression varies under different stress conditions [24,25,26]. Several novel reference genes were ranked in the top three in the comprehensive ranking of expression stability across multiple tissues of *F. carica* compared with the traditional genes (Figure 2). *FcPP2A* can normalize the transcript in the leaf and stem but not in the fruit. *FcYLS8* and *FcF-box* can be used as reliable reference genes in the fruit and leaf but not in the stem. Notably, none of the genes evaluated were among the top three across all tested tissues. The present study highlights the importance of selecting appropriate reference genes based on the plant tissue used for RT-qPCR analysis.

### 3.4. Validation of Selected Reference Genes Under Biotic Stress

**Figure 3:**
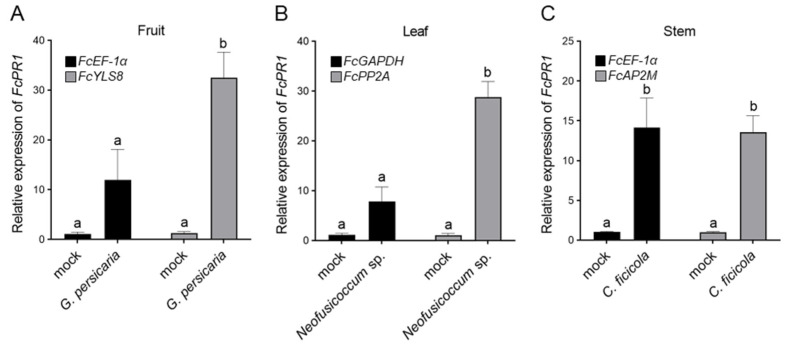
Relative expression levels of *FcPR1* in different tissues of *Ficus carica* under biotic stress. Total RNA was isolated from the fruit, leaf, and stem tissues of *F. carica* inoculated with fungal pathogens or mock. The expression levels of *FcPR1* were determined by RT-qPCR and normalized to those of reference genes, including the most and least stable genes in fruit (**A**), leaf (**B**), and stem (**C**) tissues. Values are means ± SE of three replicates. Different letters denote statistically significant differences (*p* < 0.05, two-way ANOVA with Tukey post hoc test).

Pathogenesis-related (PR) genes play a key role in the host defense mechanism during pathogen attack. In many plant species, *PR1* orthologous genes are induced in response to biotic stress [16,27,28]. While the PR1 protein is found in *Ficus* plants [29], other defense-related genes have not been studied comprehensively. Therefore, to validate the reliability of the identified reference genes, the most and least stable genes from the comprehensive ranking were used to normalize the expression of the defense-related gene *FcPR1*. The relative expression levels of *FcPR1* were investigated in three tissues (fruits, leaves, and stems) of *F. carica* inoculated with fungal pathogens compared with those of the negative control mock. Normalization using the most stable reference genes resulted in considerable upregulation of *FcPR1* in all tissues inoculated with fungal pathogens (Figure 3). However, when the least stable reference genes were used for normalization, the expression levels of *FcPR1* in fruit and leaf were lower than those normalized by the most stable genes (Figure 3A,B). Thus, the induction of the defense gene was underestimated. In stems inoculated with the fungal pathogen, similar induction levels of *FcPR1* were observed when the most and least stable genes were used for normalization (Figure 3C). However, normalization using the most stable gene *FcAP2M* resulted in a smaller standard error than that using the least stable gene *FcEF-1α*. These results highlight the importance of selecting appropriate reference genes to avoid misinterpretation of the expression data under biotic stress.

The expression stability of reference genes has been evaluated in mulberry, a member of the *Moraceae* family, under biotic and abiotic stresses [30,31]. *PP2A* is the most stable reference gene in mulberry leaves during fungal infection and salt stress. Consistently, *FcPP2A* showed high expression stability in the leaves and stems of *F. carica*, which were inoculated with the fungal pathogens *G. persicaria* and *C. ficicola*, respectively (Figure 2). Using the most stable gene, RT-qPCR analysis provided more reliable and accurate *FcPR1* expression levels (Figure 3). Alternatively, the expression levels of target genes can be normalized using multiple reference genes, as demonstrated in previous studies [9,10]. The present study provides appropriate reference genes for the normalization of RT-qPCR data from *F. carica* under biotic stress. However, a limitation of the present study is that the RT-qPCR experiments were conducted using only three plant tissues inoculated with specific fungal pathogens. Therefore, additional validation is necessary under various stress conditions, as well as in other developmental stages or tissues. Further genome-wide identification and validation of reference genes based on transcriptome data are useful for addressing such limitations and for performing gene expression analyses in *F. carica* under different experimental conditions.

## 4. Conclusions

To the best of my knowledge, this is the first systematic study to identify and validate reference genes for RT-qPCR normalization in *F. carica*. Twelve genes were selected from the *F. carica* genome as candidate reference genes, and their expression stabilities were evaluated in different plant tissues under biotic stress using computational algorithms. Based on the comprehensive ranking of expression stability, *FcYLS8*, *FcPP2A*, and *FcAP2M* were the most stable reference genes in the fruit, leaf, and stem tissues of *F. carica*, respectively. The present study is expected to facilitate future gene expression analyses related to physiological phenomena, including development and stress responses.

## Figures and Tables

**Table 1:** Primer sets and amplification characteristics of candidate reference genes in *Ficus carica*.

Gene Name	Accession Number	Homologous Locus	Primer Sequence (5′-3′)	Amplicon Size (bp)	Amplification Efficiency (%)	R^2^
*FcACT2*	LC860565	AT3G18780	ACCACTGCTGAGCGTGAAATTG	105	108.33	0.999
			AACAGCAGAGCTTGTCTTGGC			
*FcAP2M*	LC860566	AT5G46630	AATCTTCCATTCCGCGTGTTGC	117	93.47	0.997
			AATGACAACCCCAAGCGCAAAC			
*FcEF-1α*	LC860567	AT5G60390	AAGAACGGTGATGCTGGGTTTG	122	93.18	0.998
			ACAGCCACAGTTTGACGCATG			
*FcEIF4A1*	LC860568	AT3G13920	TTGCACCAACTCGAGAACTTGC	96	95.81	0.999
			TTCCACCAACACAAGCATGCAC			
*FcF-box*	LC860569	AT5G15710	AATGCTCGGTGTTTAGCTTGCC	89	107.03	0.997
			AGAACCAACCAACCAGAATGCC			
*FcGAPDH*	LC860570	AT1G13440	AAGGCTGTTGGAAAGGTTCTGC	143	93.95	0.999
			ATGGCGGCTTTCACATCTTCG			
*FcPP2A*	LC860571	AT1G13320	TTGGCTGTTGAAAGCTGTGCAG	122	100.17	0.996
			ACCATGTAACGAACACGCCATG			
*FcSAND*	LC860572	AT2G28390	AGTTGGAACCCAGCCACATTTC	83	98.10	0.997
			AATATGGCACCTGCTGCTTGC			
*FcTIP41*	LC860573	AT4G34270	CGTTTGATGCGCTGATTGGATG	84	96.06	0.999
			TGCATCGGAATTTCCACTGTGC			
*FcUBC21*	LC860574	AT5G25760	TATTGCTTTGATGGCCCATCCG	135	99.89	0.999
			TTTTTAGGCATGGCAGCAAGC			
*FcUBQ5*	LC860575	AT3G62250	ACACCATCGACAATGTGAAGGC	85	96.32	0.998
			TGCTTTCCGGCAAAGATCAGAC			
*FcYLS8*	LC860576	AT5G08290	ATCAGGAACCTCTGTGATGTCGAC	80	91.14	0.998
			ACGAAGTTCTGGCATCAGTTGC			

## Data Availability

The original contributions presented in this study are included in the article/Supplementary Material. Further inquiries can be directed to the corresponding author.

## Supplementary Materials

The following supporting information can be downloaded at: https://www.mdpi.com/article/10.3390/plants15010040/s1, Figure S1: PCR amplification specificity of the candidate reference genes used for RT-qPCR analysis; Table S1: Primer sets for cloning of candidate reference genes in *Ficus carica*.

## Abbreviations

The following abbreviations are used in this manuscript:

18S rRNA	18S ribosomal RNA
ACT	Actin
AP2M	Adaptor protein-2 μ-adaptin
EF-1	Elongation factor-1
EIF4A1	Eukaryotic translation initiation factor 4A-1
F-box	F-box protein
GAPDH	Glyceraldehyde-3-phosphate dehydrogenase
PDA	Potato dextrose agar
PP2A	Protein phosphatase 2A
RT-qPCR	Quantitative real-time PCR
SAND	SAND family protein
TIP41	TIP41-like protein
TUB	Tubulin
UBC	Ubiquitin-conjugating enzyme
UBQ	Polyubiquitin
YLS8	Yellow-leaf-specific protein 8
