# Supplementary material for: Identification and Validation of Reference Genes for Quantitative Real-Time PCR in Ficus carica
Source: Plants (Basel). 2025 Dec 22;15(1):40. doi: 10.3390/plants15010040 (PMC12788024; doi:10.3390/plants15010040)
Supplement: Supplementary file 1 [file plants-15-00040-s001.zip › plants-3995538-supplementary.pdf]

Supporting Information

Table S1. Primer sets for cloning of candidate reference genes in *Ficus carica*.

Gene symbol	Annotation	Arabidopsis homologous locus	Identity (%)	Primer sequence (5'-3')
FcACT2	Actin 2	AT3G18780<br>2093954	92	GAGGATATTCAGCCACTCGTCTGTGAC<br>GCCAGACTCGTCGTACTCTGCCTTT
FcAP2M	Clathrin adaptor complexes medium subunit family protein	AT5G46630<br>2178500	94	ATGCCGGTGGCTGCTTCCGCCGTCTA<br>AGAAAACGGACACGGAGACCCGATGCTG
FcEF-1α	Alpha subunit of the elongation factor-1 complex	AT5G60390<br>2175118	96	ATGGGTAAGGAGAAGTTTCACATCAACAT<br>TCACTTGCCACCCTTCTTGGCAGC
FcEIF4A1	Eukaryotic translation initiation factor 4A-1	AT3G13920<br>2088237	93	CAAGATTTCTTTACAACGTATGATGAAGTC<br>TCACAGGAGATCGGCAACGTTCGAC
FcF-box	Galactose oxidase/kelch repeat superfamily protein	AT5G15710<br>2143256	90	AGAAACACGAGTCCGTCAAGGCAGAA<br>ATTATCCTGTCCAAAGCACTCGAACCTCTC
FcGAPDH	Glyceraldehyde-3-phosphate dehydrogenase C2	AT1G13440<br>2010007	73	TCAAGATCGGAATCAACGGATTTGG<br>TGTAGCCCCACTCGTTGTCATACCA
FcPP2A	Protein phosphatase 2A	AT1G13320<br>2205354	90	ATGTCTTCGGGAGACGAGCCACTCTAC<br>CTAGCTAGACATCATGACATGCTCAATAGC
FcSAND	SAND family protein	AT2G28390<br>2057552	77	GTCATGGAGAAAAAGGAAGAAGCATTTCTTCAT<br>TTTTCATCTCTTCTAAATTGGGTCTTGTGGGG
FcTIP41	TOR signaling pathway protein	AT4G34270<br>2116169	72	ATGGAAGTGGAAGTCGACGAAAACGAC<br>TTAAATATGAGCAGGGACTTTAAGCACTTC
FcUBC21	Ubiquitin-conjugating enzyme 21	AT5G25760<br>2145269	94	GCATCGAGGGCGAGGCTATTCAAAGAATA<br>CTAGCCTTTTTTAGGCATGGCAGCAAGCC
FcUBQ5	Ubiquitin 5	AT3G62250<br>2098003	96	ATGCAGATCTTCGTGAAAACCCTAACGGG<br>AATCGCCTCCAGCCTTCTGATAAACGTAGG
FcYLS8	Dim1 homolog protein	AT5G08290<br>2150768	99	ATGTCGTACTTGCTGCCACACCTGCAC<br>TTAGTAGCGGTACTTGGTGGAGTAATCC

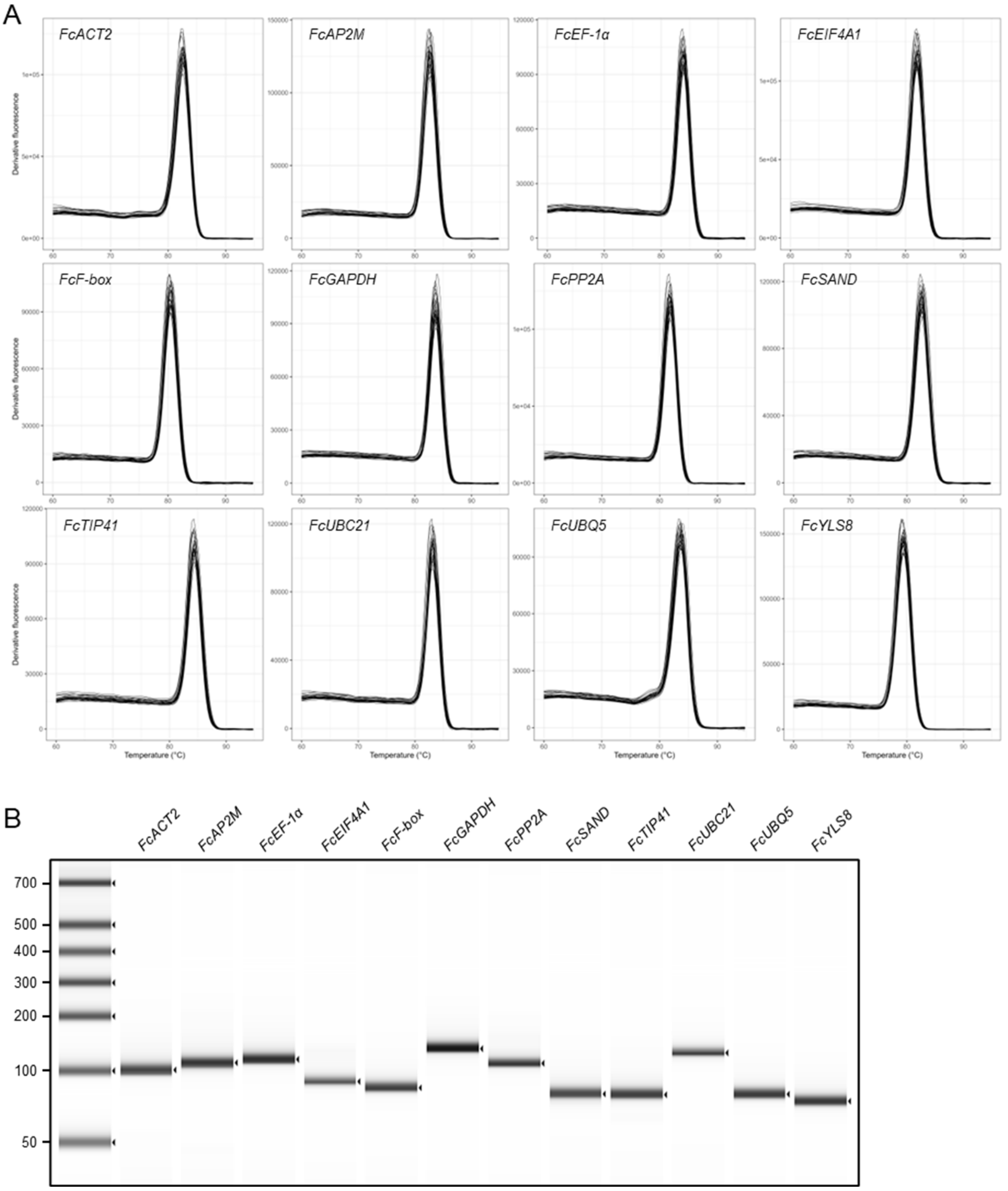

Figure S1. PCR amplification specificity of the candidate reference genes used for RT-qPCR analysis. Total RNA was isolated from fruit, leaf, and stem tissues of *Ficus carica*. The expression levels of 12 candidate reference genes were analyzed using a cDNA pool obtained from different tissues as the template for RT-qPCR. (A) Melting curves of candidate reference genes. The melting temperature of PCR amplification was measured after the RT-qPCR reaction. (B) Gel electrophoresis of candidate reference genes. The amplification products were electrophoresed after the RT-qPCR reaction.
